# Analysis of the Genome of the Sexually Transmitted Insect Virus *Helicoverpa zea* Nudivirus 2

**DOI:** 10.3390/v4010028

**Published:** 2012-01-06

**Authors:** John P. Burand, Woojin Kim, Claudio L. Afonso, Edan R. Tulman, Gerald F. Kutish, Zhiqiang Lu, Daniel L. Rock

**Affiliations:** 1 Department of Plant, Soil and Insect Sciences, University of Massachusetts Amherst, Amherst, MA 01003, USA; Email: wjkim@psis.umass.edu; 2 Plum Island Animal Disease Center, Agricultural Research Service, U.S. Department of Agriculture, Greenport, NY 11944, USA; Email: claudio.afonso@ars.usda.gov (C.L.A.); edan.tulman@uconn.edu (E.R.T.); gkutish@netzero.net (G.F.K.); zhiqiang.lu@dhs.gov (Z.L.); dlrock@illinois.edu (D.L.R.)

**Keywords:** nudivirus, sterile insects, baculovirus, sexually transmitted virus, virus genome, juvenile hormone esterase, HzNV-2, HzNV-1, corn earworm, *Helicoverpa zea*

## Abstract

The sexually transmitted insect virus *Helicoverpa zea* nudivirus 2 (HzNV-2) was determined to have a circular double-stranded DNA genome of 231,621 bp coding for an estimated 113 open reading frames (ORFs). HzNV-2 is most closely related to the nudiviruses, a sister group of the insect baculoviruses. Several putative ORFs that share homology with the baculovirus core genes were identified in the viral genome. However, HzNV-2 lacks several key genetic features of baculoviruses including the late transcriptional regulation factor, LEF-1 and the palindromic *hrs*, which serve as origins of replication. The HzNV-2 genome was found to code for three ORFs that had significant sequence homology to cellular genes which are not generally found in viral genomes. These included a presumed juvenile hormone esterase gene, a gene coding for a putative zinc-dependent matrix metalloprotease, and a major facilitator superfamily protein gene; all of which are believed to play a role in the cellular proliferation and the tissue hypertrophy observed in the malformation of reproductive organs observed in HzNV-2 infected corn earworm moths, *Helicoverpa zea*.

## 1. Introduction

*Helicoverpa zea* nudivirus 2 (HzNV-2) was originally identified as being responsible for the sterility of infected corn earworm moths (*H. zea*) in a colony of this insect established and maintained at Stoneville MS (USA). HzNV-2 [a.k.a. gonad specific virus (GSV), Hz-2V] is an enveloped, rod‑shaped, double stranded DNA (dsDNA) virus which is a member of the newly described *Nudivirus* genus [1]. Other members of this group include: the *Oryctes rhinoceros* nudivirus (OrNV), *Gryllus bimaculatus* nudivirus (GbNV) and the nudivirus (HzNV-1), initially identified in a cell line established from ovarian tissues of *H. zea* moths. Although the nudiviruses (NV) are considered a loosely associated group of non-occluded insect pathogenic dsDNA viruses, the recent analysis of OrNV genome has provided a foundation for common elements of some of the disparate members of this group [2,3]. In addition, this work describes possible evolutionary linkages between these viruses and other large dsDNA viruses of arthropods including the baculoviruses, the hytrosaviruses and the nimaviruses. Significant homology has also been found between several nudivirus genes and genes coding for both nucleocapsid proteins of bracoviruses and genes involved in the replication of these polydnaviruses. These findings have led to the suggestion that polydnavirus originated from the integration of a nudivirus ancestor into the genome of a braconid wasp [4].

Our initial characterization of the HzNV-2 genome revealed that this virus shared a 93% sequence identity with HzNV-1 [1]. This was an interesting finding in view of the fact that, while both viruses can replicate in tissue culture cells, only HzNV-2 has the ability to replicate in an insect host. In as much as the replication of either of these two viruses in cell culture can lead to establishment of persistently infected cell lines, persistent replication of HzNV-2 in the insect host, results in fertile *H. zea* moths, which are asymptomatic carriers of the virus. Productive replication of HzNV-2 on the other hand appears to be limited to the reproductive tissues of the infected moth resulting in the malformation of these tissues and sterility of the infected host. These infected moths then serve to transmit the virus to other moths during mating attempts making HzNV-2 the causative agent of the only known sexually transmitted viral diseases of insects. 

HzNV-2 infected female moths lack some reproductive structures including, ovaries, bursa copulatrix, accessory glands and spermatheca, and have grossly deformed, enlarged common and lateral oviducts, which appear as a large “Y-shaped” structure. This is a condition which has been referred as being agonadal [5,6]. In both pupae and adult females, the branches of the “Y-shaped” structure are much larger than the corresponding normal, lateral oviducts. EM observations of the branch structure in 7-day-old, infected pupae shows a high level of virus replication in cells within this structure. Viral replication in these structures results in hypertrophy of the oviducts and proliferation of cells that make up these tissues [6]. In female reproductive tissues, virus replication culminates in the formation of a “viral plug” which serves to protract the mating behavior of these females, as well as serve as a source of contaminating virus for males attempting to mate [7]. In addition, infected females produce five to seven times more sex pheromone than uninfected females and attract twice as many male mates [8].

The ability of HzNV-2 to manipulate molecular processes of the host, particularly the massive cellular and tissue transformations observed in oviducts of infected females, suggests this virus is able to direct the alteration of the physiology and gene expression in infected host tissues. In order to better understand the evolutionary relationship between HzNV-1 and HzNV-2, and to begin to identify viral genes responsible for the pathology and unique biology of this insect pathogenic virus, we undertook the sequencing of the genome of HzNV-2. 

## 2. Results and Discussion

### 2.1. Genome Features

The genome of HzNV-2 was found to be a circular dsDNA molecule of 231,621 bp. This is in good agreement with the previous restriction endonuclease size estimate of 225 to 235 kb [9], making it the largest of the insect viruses with dsDNA genomes. 

**Figure 1:**
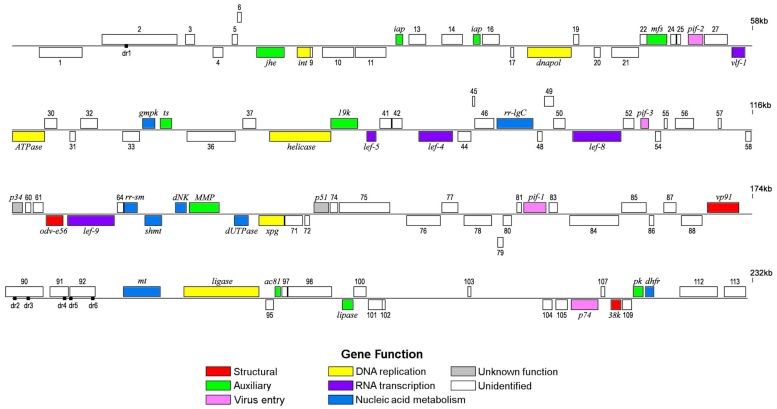
Genome map of HzNV-2. For descriptive purpose, circular genomic DNA is presented in linear form. The position of the first nucleotide was arbitrarily chosen. ORFs are numbered from left to right based on the position of the methionine initiation codon. ORFs transcribed to the right are located above the horizontal line; ORFs transcribed to the left are below.

The HzNV-2 genome has a G+C content of 41.9% and contains 376 ORFs coding more than 60 aa, of which 113 ORFs are likely to encode proteins. Of these putative genes, 66 ORFs are found on the forward strand and 47 on the reverse, all arranged evenly, producing 29 clusters of 1 to 6 ORFs in the viral genome. With sizes ranging from 5.7 kb to 189 bp, the average identified ORF was 1.4 kb in length. The gene density was one gene per 2.05 kb, and the coding density was 68%. The location, size, and direction of these ORFs is shown in Table 1 and Figure 1. For descriptive purposes, the HzNV-2 genome map is presented in linear form, and gene homologues of HzNV-1 are excluded (from Table 1, with the exception of HzNV-1 p34 and p51 late proteins which were the best blast matches) because of their high sequence homologies, which were over 90% in most cases. HzNV-1 and other nudivirus homologues are also listed in Table 1.

Unlike the baculoviruses, which have clusters of repeated, imperfect palindromic sequences, the so‑called homologous regions (*hrs*) [10], no *hrs* were found in HzNV-2 genome. No *hrs* have been found thus far in any of the other NV, including HzNV-1 [11], and GbNV [12]. The *hrs* found in baculoviruses serve as origins of viral DNA replication [13], and all of the members of *Baculoviridae* family sequenced to date have *hrs* as a common feature of their genome. Although no *hrs* were identified in the HzNV-2 genome, abundant direct tandem repeated sequences were present. Six of the most significant direct repeats (*drs*) regions of 24 to 81 bp, which repeat 7 to 25 times (Table 2), were found by EMBOSS Etandem software. Five of the six *drs* in the HzNV-2 genome form a cluster at the 174.2–180 kb region. 

In a search of GenBank database release 166, only 38 of the 113 putative HzNV-2 genes demonstrated clear homologies to genes in the current database other than putative HzNV-1 genes. These include six putative genes involved in DNA replication, four in transcription, five in nucleic acid metabolism, three structural proteins, and 14 with other functions. Hz2V058 and Hz2V073 had high homologies to the two HzNV-1 late genes, p34 and p51, respectively. The other 75 of 113 ORFs had poor or no homology to any known genes.

**Table 1:** HzNV-2 putative ORFs.

ORF	Strand *^a^*	Position		Length		Best Blast match			Signature *^b^*	HzNV-1 *^c^*
		start	end	(nt)	(aa)	Predicted function	Species/Viruses	E-value		
Hz2V001	R	5630	2298	3333	1111				TAF, H3	1
Hz2V002	F	7183	13047	5865	1955					154
Hz2V003	F	13677	14378	702	234					151
Hz2V004	R	16594	15830	765	255					150
Hz2V005	F	17366	17803	438	146					148
Hz2V006	F	17779	18063	285	95					
Hz2V007	R	21403	19241	2163	721	Carboxylesterase	*Drosophila pseudoobscura*	3 × 10^−21^	Coesterase	145
Hz2V008	R	23425	22421	1005	335	Integrase	Monodon baculovirus	1 × 10^−29^	INT_REC_C	144
Hz2V009	R	23621	23430	192	64					143
Hz2V010	R	26839	24425	2415	805					141
Hz2V011	R	29383	26969	2415	805					140
Hz2V012	F	30140	30700	561	187	Inhibitor of apoptosis protein	*Trichoplusia ni SNPV*	7 × 10^−25^	BIR	138
Hz2V013	F	31151	32494	1344	448					137
Hz2V014	F	33735	35312	1578	526					
Hz2V015	F	36168	36713	546	182	Inhibitor of apoptosis protein	*Adoxophyes orana* GV	120	BIR	135
Hz2V016	F	36879	38201	1323	441					134
Hz2V017	R	39326	39138	189	63					133
Hz2V018	R	43848	40441	3408	1136	DNA polymerase	*Oryctes rhinoceros NV*	1 × 10^−25^	POLBc	131
Hz2V019	F	44032	44442	411	137					130
Hz2V020	R	46083	45655	429	143					129
Hz2V021	R	49108	47033	2076	692					128
Hz2V022	F	49210	49731	522	174					
Hz2V023	F	49772	51307	1536	512	Membrane transporter	*Ades aegyptii*	1 × 10^−135^	MFS_1	126
Hz2V024	F	51575	52000	426	142					125
Hz2V025	F	52066	52392	327	109					124
Hz2V026	F	52998	54137	1140	380	Per-os infectivity factor 2	*Gryllus bimaculatus* NV	5 × 10^−37^	Baculo_44	123
Hz2V027	F	54224	56023	1800	600					122
Hz2V028	R	57374	56376	999	333	Very late expression factor 1	Monodon baculovirus	1 × 10^−8^		121
Hz2V029	R	60667	58199	2469	823	DNA repair related ATPase	*Gryllus bimaculatus* NV	3 × 10^−4^	SbcC	119
Hz2V030	F	60709	61674	966	322					118
Hz2V031	R	63063	62671	393	131					117
Hz2V032	F	63532	64803	1272	424					115
Hz2V033	R	68086	66803	1284	428					112
Hz2V034	F	68319	69323	1005	335	Guanosine monophosphate kinase	Monodon baculovirus	4 × 10^−5^	GMPK	111
Hz2V035	F	69749	70621	873	291	Thymidylate synthase	*Bombyx mori*	4 × 10^−131^	Thymidylat_synt	109
Hz2V036	R	75567	71800	3768	1256					107
Hz2V037	F	76171	77190	1020	340					106
Hz2V038	R	83046	78304	4743	1581	Helicase	*Culex nigripalpus* NPV	2.8	Pox_D5	104
Hz2V039	F	83048	85156	2109	703	19kDa protein	*Gryllus bimaculatus* NV	9 × 10^−9^	Baculo_19	103
Hz2V040	R	86584	85862	723	241	Late expression factor 5	*Spodoptera exigua* NPV	6 × 10^−3^	Baculo_LEF5	101
Hz2V041	F	86863	87756	894	298					100
Hz2V042	F	87845	88594	750	250					99
Hz2V043	R	92546	89928	2619	873	Late expression factor 4	*Gryllus bimaculatus* NV	3 × 10^−18^		98
Hz2V044	R	94003	92963	1041	347					97
Hz2V045	F	94110	94313	204	68					
Hz2V046	F	94306	95775	1470	490					96
Hz2V047	F	96009	98822	2814	938	Ribonuclease reductase	*Spodoptera litura* NPV	0	Ribonuc_red_lgC	95
Hz2V048	R	99573	99214	360	120					94
Hz2V049	F	99730	100473	744	248					93
Hz2V050	F	100458	101336	879	293					
Hz2V051	R	105733	101939	3795	1265	Late expression factor 8	*Gryllus bimaculatus* NV	1 × 10^−12^	RNA_pol_Rpb2_6	90
Hz2V052	F	105870	106712	843	281					89
Hz2V053	F	107253	107894	642	214	Per-os infectivity factor 3	*Gryllus bimaculatus* NV	2 × 10^−10^		88
Hz2V054	R	108853	108413	441	147					87
Hz2V055	F	109110	109391	282	94					85
Hz2V056	F	109953	111365	1413	471					83
Hz2V057	F	113318	113530	213	71					
Hz2V058	R	115909	115496	414	138					81
Hz2V059	F	116202	116963	762	254	p34 late protein	*Helicoverpa zea* NV-1 ^d^	8 × 10^−147^		79
Hz2V060	F	117224	117595	372	124					78
Hz2V061	F	117803	118570	768	256					77
Hz2V062	R	120105	118786	1320	440	Odv-e56 structural protein	*Gryllus bimaculatus* NV	2 × 10^−9^		76
Hz2V063	R	124121	120513	3609	1203	Late expression factor 9	Monodon baculovirus	1 × 10^−60^		75
Hz2V064	F	124357	124818	462	154					74
Hz2V065	F	124932	125930	999	333	Ribonuclease reductase	*Xenopus tropicalis*	5 × 10^−128^	Ribonuc_red_sm	73
Hz2V066	R	127832	126510	1323	441	Serine hydroxymethyltransferase	*Bombyx mori*	0	SHMT	72
Hz2V067	F	128939	129760	822	274	Deoxynucleotide kinase	*Drosophila melanogaster*	1 × 10^−38^	dNK	71
Hz2V068	F	129972	132338	2367	789	Matrix metalloprotease	*Acyrthosiphon pisum*	3 × 10^−69^	ZnMc_MMP, HX	70
Hz2V069	R	134585	133536	1050	350	dUTPase	*Culex quinquefasciatus*	2 × 10^−38^	dUTPase	69
Hz2V070	R	137400	135430	1971	657	DNA excision repair enzyme	*Musca domestica* SGHV	2 × 10^−7^	XPG	68
Hz2V071	R	138863	137478	1386	462					67
Hz2V072	R	139354	139019	336	112					66
Hz2V073	F	139721	140869	1149	383	p51 late protein	*Helicoverpa zea* NV-1 ^d^	0		64
Hz2V074	F	141013	141564	552	184					63
Hz2V075	F	141693	145637	3945	1315					62
Hz2V076	R	149608	146981	2628	876					60
Hz2V077	F	149749	150978	1230	410					59
Hz2V078	R	153629	151443	2187	729					58
Hz2V079	R	154524	154090	435	145					
Hz2V080	R	155191	154481	711	237					
Hz2V081	F	155556	155936	381	127					56
Hz2V082	F	156150	157853	1704	568	Per-os infectivity factor 1	*Gryllus bimaculatus* NV	6 × 10^−23^	DUF686	55
Hz2V083	F	158080	158742	663	221					54
Hz2V084	R	163557	159694	3864	1288					52
Hz2V085	F	163804	165702	1899	633					51
Hz2V086	R	166298	165954	345	115					
Hz2V087	F	167038	168003	966	322					49
Hz2V088	R	170084	168429	1656	552					47
Hz2V089	F	170456	172948	2493	831	vp91 capsid protein	*Gryllus bimaculatus* NV	4 × 10^−14^		46
Hz2V090	F	173649	176570	2922	974					
Hz2V091	F	177136	178524	1389	463					
Hz2V092	F	178677	180614	1938	646					
Hz2V093	F	182846	185689	2844	948	Methyltransferase	*Helicoverpa zea* NPV	8 × 10^−28^	FtsJ	37
Hz2V094	F	187572	193430	5859	1953	DNA ligase	*Apis mellifera*	4 × 10^−86^	DNA_ligase_A_M	36
Hz2V095	R	194548	193964	585	195					34
Hz2V096	F	194663	195130	468	156	Ac81	*Gryllus bimaculatus* NV	6 × 10^−12^	DUF845	33
Hz2V097	F	195232	195630	399	133					32
Hz2V098	F	195699	199133	3435	1145					31
Hz2V099	R	200794	199931	864	288	Esterase/lipase	*Psychromonas ingrahamii*	0.16	Aes	30
Hz2V100	F	200858	201796	939	313					29
Hz2V101	R	203059	201992	1068	356					28
Hz2V102	R	203298	203092	207	69					
Hz2V103	F	209783	209980	198	66					
Hz2V104	R	216330	215599	732	244					13
Hz2V105	R	217543	216668	876	292					12
Hz2V106	R	219936	217846	2091	697	p74 envelope protein	*Gryllius bimaculus* NV	5 × 10^−57^	Baculo_p74	11
Hz2V107	F	220177	220464	288	96					
Hz2V108	R	221727	220951	777	259	38kDa protein	Monodon baculovirus	3 × 10^−15^	DUF705	10
Hz2V109	R	222547	221819	729	243					9
Hz2V110	F	222677	223438	762	254	Protein kinase	*Trichomonas vaginalis*	5 × 10^−18^	S_TKc	8
Hz2V111	F	223656	224297	642	214	Dihydrofolate reductase	*Heliothis virescens*	1 × 10^−45^	DHFR	7
Hz2V112	F	226341	229232	2892	964					4
Hz2V113	F	229783	231468	1686	562					3

*^a^* The direction of the transcripts are indicated by F (forward) and R (reverse); *^b^* Functional motif found in Pfam and CDD database; *^c^* HzNV-1 ORFs corresponding to that of HzNV-2; ^d^ HzNV-1 ORFs which are the best match to that of HzNV-2.

**Table 2:** HzNV-2 direct repeat (*drs*) sequences.

	start	end	score	size	Count	%id	consensus
dr1	11711	12310	134	24	25	63.2	atgaagctgaggatgaatctgaac
dr2	174285	174572	132	36	8	79.2	gaaactcctaaatcaaaggatgaa cctaaagcaaag
dr3	175417	175656	124	60	4	88.3	atgaaaaagcaaaggctgaggcga aggctaaagccgatgctgctgcaa aagccaaagctg
dr4	178103	178390	182	24	12	85.8	ttataccagagagcaagccagaaa
dr5	178517	178921	104	81	5	72.8	acctaaagttgaatctaaagtagt ggaaccacctaaagcggaatctaa aacagtggaagctcctactaaaac agttgaagt
dr6	180236	180445	156	30	7	94.3	agctgccgctaaacgcaaagccg aggctga

Direct repeat sequences were found by using EMBOSS Etandem software with a cutoff score of 100.

**Figure 2:**
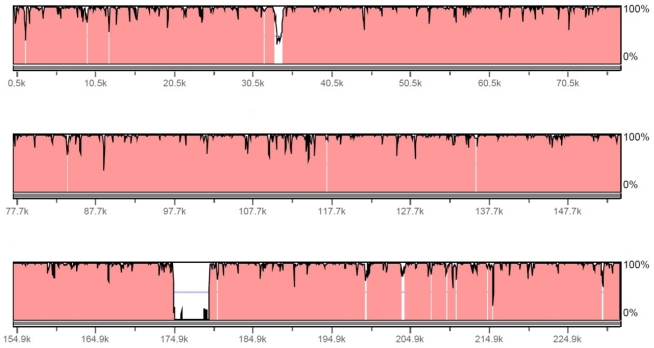
Comparison of HzNV-1 and HzNV-2 genome sequence. The 228089 bp of the HzNV-1 genome sequence was subjected to rVISTA software to compare with 231621 bp of the HzNV-2 genome sequence. HzNV-2 is the base sequence, the sequence identity is plotted in 0~100% scale, and a region with 95% or higher identity is presented in red. The sequence window size is 100 bp.

### 2.2. Evaluation of ORFs

Within the HzNV-2 genome sequence, a total of 376 virtual ORFs were identified that were longer than 60 aa and started with a methionine initiation codon. Minor ORFs, which were completely included within larger ORFs, were excluded. A total of 163 ORFs with a Glimmer score greater than 90 points were found among the 376 putative ORFs and were subjected to further evaluation. Hexamer scores were used to exclude ORFs with a low coding potential, and to eliminate ORFs which had a significant overlap with a portion of adjacent ORFs on either the same or complimentary strand. A total of 113 ORFs among the 163 ORFs that passed the GLIMMER cutoff were selected for database search and physical characterization to identify possible functions of the putative genes.

In the initial analysis of the viral genome, it became clear that HzNV-2 was very closely related to the insect virus HzNV-1. Gene parity plot results demonstrated that 97 out of 113 putative HzNV-2 ORFs were found in the HzNV-1 genome without any overlap or inversion (Table 1). The global DNA sequence alignment between HzNV-1 and HzNV-2 by EMBOSS Matcher and rVISTA software showed 93.5% sequence identity with a 5.1 kb area at 174.7–179.8 kb region of HzNV-2 that showed significant gaps in the corresponding region of HzNV-1 genome (Figure 2). There were 3 ORF found in region: Hz2V090, Hz2V091, and Hz2V092. There were a total of 14 ORFs identified in the HzNV‑2 genome that were not found in the HzNV-1 genome. None of these ORFs were determined to share sequence homology with any genes of know function. 

The Genbank accession number of the Hz-2V genome sequence is JN418988.

### 2.3. Enzymes Related to DNA Replication and Repair

Five putative genes, Hz2V008 (integrase), Hz2V018 (dnapol), Hz2V029 (ATPase), Hz2V038 (helicase), Hz2V070 (excision\repair), and Hz2V094 (ligase) were orthologues of genes likely involved in DNA replication and repair. The C-terminal region of Hz2V008 had homology to the catalytic domain for integrase/recombinase, which has DNA breaking-rejoining activity. This region of Hz2V008 had relatively good similarity (E = 2 × 10^−9^) to the INT_REC_C conserved domain, which is related to phage integrase and bacterial and yeast recombinase. It contained well-conserved functional motif cores for DNA binding sites, catalytic residues, the active sites, and topoisomerase. The integrases from monodon baculovirus (MBV) and GbNV showed the best similarity (E = 1 × 10^−29^ and 2 × 10^−11^, respectively) to Hz2V008 suggesting a possible common ancestral origin for these viral genes. Hz2V008 did not show homology with any baculovirus genes.

Hz2V018 had homology to DNA polymerase. The C-terminal region of Hz2V018 had (E = 7 × 10^−10^) similarity to the DNA dependent DNA polymerase type-B family (POLBc) conserved domain, which has DNA binding, polymerase, and 3'-5' exonuclease activity [14]. In this region, the well-conserved nucleotide binding motif, K(3x)NS(x)YG(2x)G, and the polymerase catalytic motif, YxDTD, were found at position 842–853 aa, and 892–897 aa, respectively. Hz2V018 also showed homology to the OrNV DNA polymerase. 

Hz2V029 is an 823 aa long protein, with 256 aa at the N-terminal region which showed similarity (E = 2 × 10^−3^) to the C-terminal region of the SbcC conserved domain. The SbcC gene is a prokaryotic ortholog of the rad50 gene of humans that has ATPase activity and plays an important role in DNA double-strand break repair [15,16]. The SbcC gene is known to play a similar role during DNA replication in prokaryotes [17]. The highest similarities were detected for GbNV putitive desmoplakin (E = 3 × 10^−4^) and *Spodoptera frugiperda* ascovirus 1a SbcC/ATPase domain (E = 4 × 10^−4^).

Hz2V038 had homology to DNA helicase and was identified by the presence of a poxvirus D5 protein-like domain (Pox_D5) at its C-terminal region), [18]. Helicase is an essential gene for virus replication, and all baculoviruses contain a conserved helicase domain. 57.9% of residues of the conserved helicase domain aligned to over 186 aa of the Hz2V038 C-terminal region at position 1138–1323 aa, however, this similarity was relatively low (E = 4 × 10^−3^). *Culex nigripalpus* helicase-1 was detected by a BLASTP search with Hz2V038, but with a poor score (E = 2.3). Hence PSI-BLAST was used to detect homologous genes. In the second iteration of PSI-BLAST, homologies to helicase genes of various baculoviruses were detected near the region of Pox_D5 domain. The *Ectoropis oblique* nucleopolyhedrovirus (NPV) helicase showed highest homology.

Hz2V070 had homology to xeroderma pigmentosum G (XPG) enzyme, which is related to other enzymes involved in nucleotide-excision repair and transcription-coupled repair of oxidative DNA damage. XPG has been shown to function by making a nick at the 3' side of the DNA strand damaged by UV radiation, which is then mended via the DNA excision repair system [19,20]. XPG is functionally and structurally related to flap endonuclease (FEN-1) enzyme [21], which functions in a manner analogous to the base excision DNA repair mechanism [22]. The homology between Hz2V070 and XPG was detected in the N-terminal region at position 28–256 aa, and CD-Search detected that the XPG domain had 245 of 319 residues aligned to the N-terminal region of Hz2V070 with relatively low similarity (E = 6 × 10^−3^). Because of the subtle homology of Hz2V070 to the XPG domain, BLASTP was able to detect only three homologues, found in genomes of the insect pathogenic, DNA viruses GbNV, OrNV and the salivary gland hypertrophy of *Musca domestica*, (MdSHGV), when the search was limited to the database subset of virus sequences.

Hz2V094 codes for a large putative protein composed of 1953 aa. This putative gene product had a highly conserved (E = 3 × 10^−46^) ATP-depentent DNA ligase domain of 201 aa in length that had high similarity to the DNA_Ligase_A_M conserved domain. The best match was to *Apis melifera* DNA ligase III (E = 5 × 10^−84^). The DNA ligase catalytic domain was located at the end of C-terminal region of Hz2V094. It also showed homology to other ligases at positions 480–714 aa and 1541–1929 aa regions. There was a more than 800 aa insertion between these two regions that did not show homology to any other genes. 

### 2.4. Proteins Involved in RNA Transcription

There are five putative genes, Hz2V028 (*vlf-1*), Hz2V040 (*lef-5*), Hz2V043 (*lef-4*), Hz2V051 (*lef‑8*), and Hz2V063 (*lef-9*), identified in the HzNV-2 genome that shared homology with baculovirus genes known to be involved in transcription. This is a very interesting result since these genes are all conserved baculovirus core genes that function in transcription and expression of late and very late genes. Baculoviruses share six common genes related to transcription, *p47*, *lef-4*, *lef-5*, *lef-8*, *lef-9*, and *vlf-1* [23]. The recent analysis of the OrNV genome sequence identified all six of these core genes in the genome of this virus, as well as in the genome of HzNV-1, and GbNV [2]. Our analysis identified all of these genes as well, except for *p47*. 

These five common genes can be categorized into two groups. Both *lef-5* and *vlf-1* are transcription initiation factors, while *lef-4*, *lef-8* and *lef-9* along with *p47* encode subunits of RNA polymerase [24]. Hz2V028 showed homology to baculovirus very late expression factor 1 (vlf-1), which is essential for burst expression of the very late baculovirus genes, *polyhedrin* and *p10* [25]. *Vlf-1* is a transcription initiation factor that recognizes and binds to the promoter motif, DTAAG, of baculovirus very late genes [26] and is also involved in capsid assembly and DNA packaging [27]. Transient expression studies with the baculovirus very late promoter motif and HzNV-1 RNA polymerase demonstrated however, that HzNV-1 does not recognize the DTAAG motif [28]. This result indicates that HzNV-2 *vlf-1*, which has a 93% aa sequence similarity with the HzNV-1 *vlf-1* homologue, possibly recognizes a different late or very late promoter motif than does the baculovirus *vlf-1*. This is not surprising since the similarity of Hz2V028 to baculovirus *vlf-1* was relatively low (E = 2 × 10^−2^). 

Hz2V040 had homology to late expression factor 5 (*lef-5*). LEF-5 has a characteristic zinc ribbon motif for DNA binding, which is often found in eukaryotic transcription elongation factors. However, LEF-5 along with baculovirus RNA polymerase, has transcriptional initiation activity at the late gene promoter [29]. Hz2V040 showed relatively low similarity to the C-terminal region of the Baculo_LEF-5 conserved domain (E = 6 × 10^−3^) at position 165–193 aa. This region also showed homology to the zinc finger motif (ZnF_C2C2) which is well conserved, especially at the positions of cysteine residues. Hence, Hz2V040 probably has a secondary structure similar to the DNA binding motif of baculovirus LEF-5 and therefore, DNA binding activity. Hz2V040 showed the best homology to *Spodoptera furugiperda* NPV *lef*-5 (E = 6 × 10^−3^). Interestingly, *lef*-5 homologues were also found in the other NV, GbNV and OrNV, however, these homologues were not detected by BLASTP because of their subtle similarities. None the less, these nudivirus homologues were detected by PSI-BLAST at their zinc ribbon domain.

The best match of Hz2V043 was to late expression factor 4 (*lef*-4) of GbNV (E = 3 × 10^−18^). LEF-4 has guanyltransferase and RNA triphosphatase activity and is thought to play a role in mRNA capping, which is essential for baculovirus replication [24,30,31]. Hz2V043 (873 aa) was almost twice as long as the homologue of *Autographa californica* NPV (AcMNPV) (464 aa), and the C-terminal region of this ORF showed homology to the *lef*-4 gene of GbNV and OrNV. Two KxDG motifs, characteristic of RNA capping enzymes capable of forming a stable enzyme-nucleotide monophosphate complex for guanylation [32], were found at positions 641 aa and 764 aa. This result supports the possibility of Hz2V043 having mRNA 5'-capping activity, despite its low similarity to baculovirus *lef-4* genes.

Hz2V051 showed homology to late expression factor 8 (*lef-8*). *Lef-8*, in conjunction with *lef-9*, encodes one of the main catalytic subunits of the baculovirus RNA polymerase. Hz2V051 showed the best similarity to GbNV *lef-8* (E = 1 × 10^−12^); however, the similarity to other baculovirus *lef-8* genes was relatively low. Hz2V051 was 1265 aa long, and the region at position 650–1112 aa was detected as an RNA_pol_Rpb2_6 conserved domain. This C-terminal region of Hz2V051 contains a conserved sequence motif GxKx4HGQ/NKG at 858–869 aa, which is found in both prokaryotic and eukaryotic RNA polymerases as well as those of the insect baculoviruses [33]. 

Hz2V063 had homology to the N-terminal region of the NV and baculovirus late expression factor 9 (LEF-9). OrNV LEF-9 showed good similarity (E = 2 × 10^−8^) at this region, however, the similarity of baculovirus LEF-9 detected by PSI-BLAST at this region was low. Hz2V063 contained an HADQDGD motif at position 286–292 aa, which is similar to the structure of the NTDCDGD motif of AcMNPV LEF-9 [34] and the NADFDGD motif of the vaccinia virus RNA polymerase large subunit [35]. LEF-9, in association with LEF 8, comprises the baculovirus RNA polymerase [36]. The exact function of LEF-9 in the polymerase, however, is not clear.

### 2.5. Genes Involved in Virus Entry

Baculoviruses also have common core genes encoding structural proteins related to their *per os* infectivity. HzNV-2 was found to have four genes, Hz2V026, Hz2V053, Hz2V082, and Hz2V106, which are homologues to these baculovirus *per os* infectivity factors (PIF). These *pif* genes are also well conserved in other NVs [1,2]. The identification of putative HzNV-2 *pif* genes is a very interesting result since, in contrast to the baculoviruses and other NVs, HzNV-2 is not highly infectious *per os* [5]. The presence of *pif* gene homologues in the HzNV-2 genome suggests a common mode of entry into cells which has been evolutionarily conserved among these different viruses.

Hz2V026 showed best similarity to GbNV *pif-2* gene (E = 5 × 10^−37^) and showed good similarity to AcMNPV *pif-2* (E = 7 × 10^−11^). The *pif-2* gene product which in addition to playing a role in oral infection by baculoviruses, is also a structural component of the occlusion-derived virus envelope [37]. Interestingly, the positions of cysteine residues were well conserved between Hz2V026 and the other *pif-2* genes. This result indicates that the HzNV-2 PIF-2 homologue might form disulfide bonds in the same manner and therefore have a very similar secondary structure to the *pif-2* gene products of these other viruses. 

Hz2V053 was found to be a homologue of the baculovirus *pif-3* gene and showed good similarity to GbNV *pif-3* gene (E = 2 × 10^−10^). Similarity to other baculovirus *pif-3* genes was low, but as with PIF-2, the positions of cysteine residues were well conserved between Hz2V053, the PIF-3 of GbNV and those of other baculoviruses [38]. Despite the low similarity to baculovirus *pif-3*, Hz2V053 does contain an N-terminal transmembrane domain at position 3-19 aa, characteristic of other PIF-3s.

Hz2V082 is a homologue of the baculovirus *pif-1* gene. This gene showed the best similarity to the GbNV *pif-1* gene (E = 6 × 10^−23^) and homology with other baculovirus *pif-1* genes at the region of 70–350 aa. PIF-1 is a viral structural protein which, along with PIF-2, is responsible for oral infectivity by mediating the direct binding of the virus particle to host cells [39]. As with the other PIFs, the position of cysteine residues in PIF-1 and Hz2V08 are well conserved. When Hz2V082 was subjected to ANTHEPROT, three transmembrane domains and a putative N-terminal signal peptide cleavage site were found. This result suggests that the localization of Hz2V082 gene product in the virus membrane is similar to that of the *Spodoptera Littoralis* NPV (SlNPV) PIF-1. However, the topology of the HzNV-2 protein in the membrane might be slightly different, since the SlNPV PIF-1, and the PIF-1 other baculovirus have an additional fourth putative transmembrane domain [40].

Hz2V106 showed homology with the bacuolvirus *p74* gene, which codes for a critical oral infection factor of baculoviruses. P74 is a highly conserved envelope protein of baculoviruses that mediates specific binding of the virus particle to host cells [41]. The best similarity found for Hz2V106 was with the GbNV *p74* gene (E = 5 × 10^−57^). The proposed secondary structure of the *p74* gene product of *Choristoneura fumiferana* granulovirus (GV) illustrates that it has a characteristic positioning of cysteine residues that allows for the formation of disulfide bonds within its C-terminal transmembrane membrane anchoring domain [42]. Hz2V106 has six cysteine residues at positions 99, 104, 138, 169, 217, and 231 with three C-terminal transmembrane domains at 494–516 aa, 639–661 aa, and 665–687 aa, which are in a very similar context to those in the highly conserved baculovirus P74 protein.

### 2.6. Enzymes Involved in Nucleic Acid Metabolism

Seven putative HzNV-2 ORFs, Hz2V035, Hz2V047, Hz2V065, Hz2V066, Hz2V067, Hz2V069, and Hz2V093, showed homology to previously identified genes from eukaryotes, prokaryotes, and viruses that coded for proteins having activity related to nucleic acid metabolism. 

Hz2V035 was a homologue of thymidylate synthase (TS). It contained the highly conserved TS functional domain and showed over 80% sequence identity to other TS genes. The HzNV-2 TS gene showed the best similarity to that of *Bombyx mori* TS (E = 4 × 10^−131^) and also showed high similarity to the TS genes of herpesvirus, NV, iridovirus, and other viruses. So far, no baculovirus TS genes have been found. TS is well conserved across species and is involved in the synthesis of dTMP precursors from dUTP [43,44]. Interestingly, all identified viral TS genes are thought to be acquired from their hosts in independent incidents rather than originating from a common ancestor [45,46].

Hz2V047 and Hz2V065 were homologues to ribonucleotide reductase large (RR1) and small subunit (RR2), respectively. These genes also showed homology to a variety of RR genes with over 70% amino acid similarities. The best match was to SlNPV RR1 (E = 0). RR1 and RR2 are involved in reducing ribonucleotides into deoxyribonucleotides to produce precursors of DNA [47] and are commonly found in viruses as well as prokaryotes and eukaryotes.

Hz2V066 showed homology to the serine hydroxymethyltransferase (SHMT) conserved domain (E = 7 × 10^−171^). SHMT catalyzes the reversible interconversion of serine and glycine with tetrahydrofolate [48]. This is the major source of one-carbon groups used in the biosynthesis of compounds containing methyl groups and purine rings, as well as in thymidylate biosynthesis [49]. Hz2V066 showed the greatest similarity to the *Bombyx mori* SHMT gene (E = 0). SHMT is a cellular gene, and to date has not been found in any other eukaryotic viruses.

Hz2V067 was homologous to deoxynucleotide kinase (dNK), with the best match to dNK of *Drosophila melanogaster* (E = 1 × 10^−38^) having 57% similarity. dNK catalyzes the phosphorylation of deoxyribonucleosides to yield corresponding monophosphates and is a key enzyme involved in the salvage of deoxyribonucleosides [50]. 

Hz2V069 is a 350 aa long ORF, of which over 150 aa of the N-terminal region showed homology with the dUTP phosphatase (dUTPase) domain (E = 1 × 10^−31^). The best match was *Culex quinquefasciatus* dUTPase (E = 2 × 10^−38^), however, this gene product is also often found as a protein component of animal viruses [51]. dUTPase provides a substrate for TS and maintains low dUTP:dTTP ratio to minimize the misincorporation of uracil into virus DNA during replication [52]. dUTPase also plays an important role during virus replication in terminally-differentiated or non‑dividing cells that have a low level of cellular dUTPase expression [53]. Hence, dUTPase might be a key enzyme related to HzNV-2 replication and latency in asymptomatic carriers.

Hz2V093 encoded a 948 aa long protein that showed homology to methyltransferase (MT) at the N‑terminal region of this protein. The best match was *H. zea* NPV ORF65 (E = 8 × 10^−28^). MT is often found in NPVs, but other than HzNV-1, it has not been found in NVs. Baculovirus methyltransferase along with LEF-4 is believed to be involved in RNA capping of late and very late gene transcripts, however, it is not essential for virus replication [54].

Hz2V111 was a homologue of dihydrofolate reductase (DHFR). The best match was *Heliothis virescens* DHFR (1 × 10^−45^), with good homology to herpesvirus DHFR. DHFR reduces dihydrofolate to tetrahydrofolate, which is required to regenerate methylenetetrahydrofolate by thymidylate synthase, an essential step in the biosynthesis of deoxythymine phosphate for DNA synthesis [55]. DHFR was also found in MdSGHV, however, it has not yet been found in any baculoviruses.

### 2.7. Structural Proteins

Hz2V062 was a homologue of the baculovirus *odv-e56* gene, which encodes an occlusion-derived virus envelope protein. It showed the best match to GbNV *odv-e56*, while the similarity to other baculovirus *odv-e56* was very low. Odv-e56 is one of the baculovirus late genes which utilize a TAAG promoter motif for its expression [56]. A TAAG motif, however, was not found within the 500 bp region upstream of Hz2V062. This observation is also indicative of the possibility that the late or very late gene regulation mechanism of HzNV-1 and HzNV-2 has evolved differently from that of baculoviruses.

Hz2V089 was a homologue of baculovirus *vp91*. The VP91 in baculoviruses is localized near the nuclear membrane of the infected cell and is associated with the virion capsid [57]. The C-terminal region of Hz2V089 showed homology to the GbNV *vp91* gene (E = 4 × 10^−14^) and also other baculovirus *vp91* genes as well.

Hz2V108 was a homologue of baculovirus 38K protein gene which codes a structural protein associated with the virus nucleocapsid [58]. The 38K protein plays an important role in nucleocapsid assembly, interacting with the other nucleocapsid proteins, VP1054, VP39, VP80, and 38K itself [58]. The best match was MBV baculovirus (3 × 10^−15^). 

### 2.8. Auxiliary and Undefined Genes

Hz2V007 codes for a putative protein composed of 721 aa. This putative gene product showed highest similarity to the *Bombyx mori* carboxylesterase (COE) (E = 4 × 10^−21^) and also had a high similarity to *Anopheles gambiae* juvenile hormone esterase (JHE) (E = 2 × 10^−19^). Hz2V007 had a COE domain at position 25–202 aa that had significant similarity to other highly conserved COE domains (E = 6 × 10^−31^). Almost the entire peptide sequence of Hz2V007 at position 1–632 aa lined up with the COE domain with relatively high similarity (E = 5 × 10^−11^). 

Juvenile hormone (JH) is produced in the corpora allata, of insects, and acts as a transcriptional regulator, controlling the expression of a variety of genes involved in physiological, developmental, and reproductive processes in insects. One pathway for the control of JH titer in insects is by degradation via JHE [59]. The high similarity between Hz2V007 and insect JHEs is a very interesting result since it suggests that HzNV-2 can control the physiology of the infected host insects by regulating JH levels and ultimately the level of gene expression in various tissues during their development, at specific times during the replication cycle of the virus. 

To compare Hz2V007 with other insect JHEs, nine JHEs peptide sequences from four different insect orders were selected from Genbank including *D.mel* COE-6, one hymenopteran (*Apis melifera*), one coleopteran (*Tenebrio molitor*), three dipterans (*Drosophila melanogaster*, *Aedes aegypti*, and *Anopheles gambiae*), and three lepidopterans (*Heliothis virecens*, *Manduca sexta*, and *Bombyx mori*). Sequence alignments of the entire coding region of each JHE with Hz2V007 were performed by EMBOSS Water software. Hz2V007 showed highest peptide sequence identity and similarity within the catalytic domain of *A. gambiae* JHE (30.1% and 43.9% respectively), and the lowest with *H. virescens* JHE (20.4% and 34.6%). This level of similarity was relatively low compared with the range of sequence identities and similarities found among the nine insects JHEs, which had identities ranged between ~28.6 and 61.2% and similarities between ~61.2 and 77.1%.

**Figure 3:**
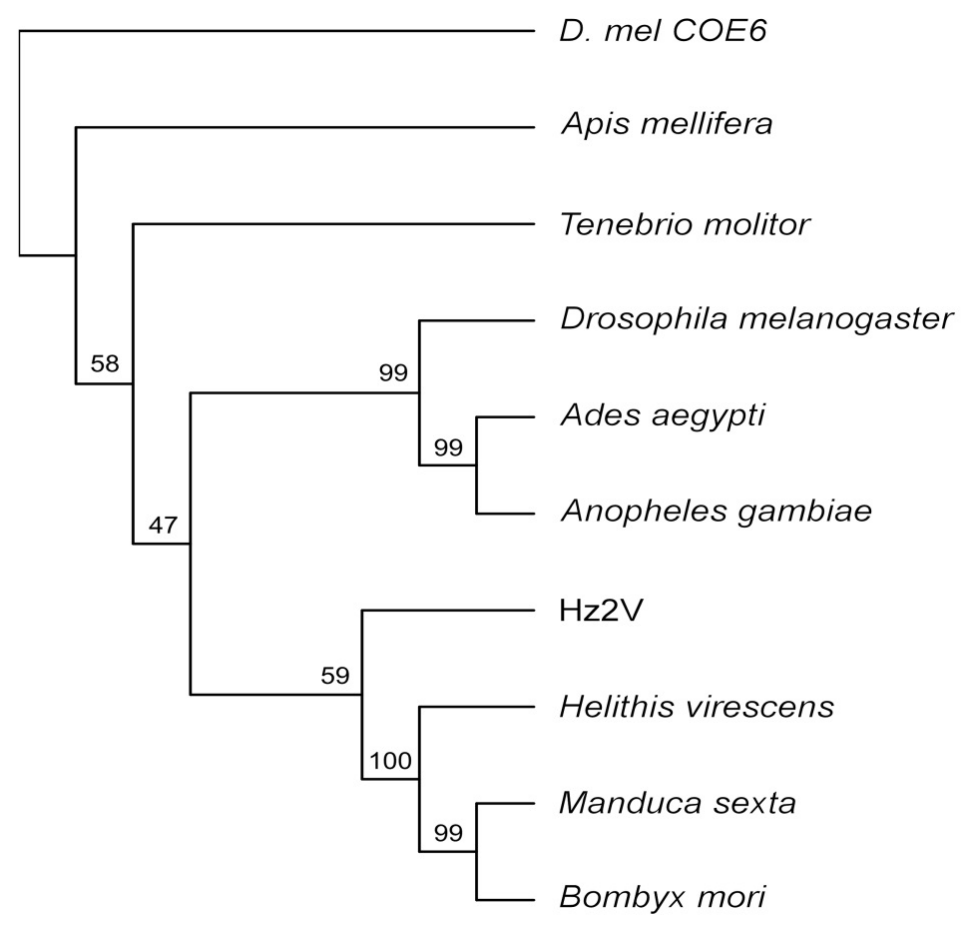
Phylogenetic analysis of JHEs and Hz2V007. Phylogenetic analysis of selected JHEs and Hz2V007. Multiple sequences were aligned using MUSCLE, and the tree was inferred using neighbor-joining in ClustalX. *D. melanogaster* corboxylesterase-6 gene was used as the outgroup. The robustness of the tree was tested using bootstrap analysis (1000 replicates). The percent values are presented at the nodes.

To investigate the phylogenetic positioning of Hz2V007 among these insect JHEs, molecular phylogenetic analysis using neighbor-joining in MUSCLE and ClustalX was performed. The results demonstrated that Hz2V007 grouped with the JHEs from lepidopteron insects, rather than to dipteran JHEs to which it had the highest sequence similarity (Figure 3). This result may reflect the closer relationship between HzNV-2 and lepidopteran insects, from which the virus was most likely to have obtained the ancestral form of Hz2V007. 

Another characteristic feature that Hz2V007 shares with other JHEs was a signal peptide. A possible cleavable signal peptide was found at position 1–20 aa, flanked by a putative cleavage site between 20 and 21 aa. The signal peptide sequences, which are responsible for secretion of insect JHE, were found in all nine JHEs.

**Figure 4:**
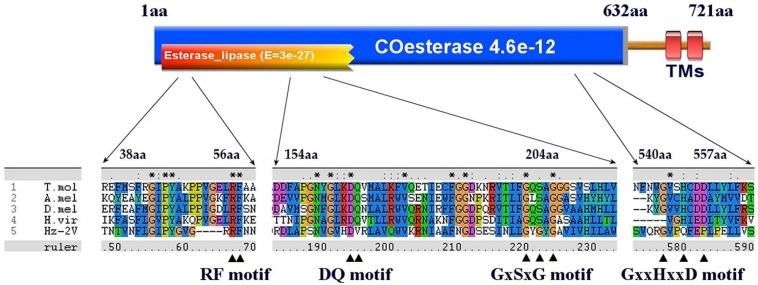
A schematic presentation of functional domains and motifs in Hz2V007. The functional domains identified by CDD and pfam database searches are presented in orange and blue, respectively. The transmembrane domains are presented in red. Multiple JHE sequence alignments are presented using ClustalX, and the locations of conserved residues in the active sites of JHE are indicated with triangles. T. mol (*T. molitor*), A.mol(*A. melifera*), D.mel (*D. melanogaster*), H.vir (*H. virecens*).

The signal peptide sequence prediction and phylogenetic analysis results alone are not sufficient to predict the JHE activity of Hz2V007. To further assess its possible JHE activity, Hz2V007 and four JHEs from each order were chosen, and multiple sequence alignment was performed to compare functional motifs within the catalytic domain of the JHEs with corresponding regions of Hz2V007. Most of the JHEs have five conserved catalytic motifs, RF, DQ, GxSxG, E, and GxxHxxD/E [60]. In Hz2V007, an intact RF motif was found at position 53 aa and partially conserved DQ, GxSxG and GxxHxxD motifs were found at 165 aa, 191 aa, and 544 aa, respectively. An E motif was not identified (Figure 4). 

The average polypeptide length of the nine JHEs is 573 aa, and the entire polypeptide sequence of JHE corresponded to the region at 1–632 aa of Hz2V007. This means that Hz2V007 has 89 aa of C‑terminal flanking sequence beyond the JHE sequence. This region showed no significant homology to any known sequences in the database. Interestingly, the SMART program predicted a transmembrane domain in this C-terminal region of Hz2V007 at position 686–719 aa, while it predicted an N-terminal signal peptide sequence for secretion. This result suggests a unique localization of the Hz2V007 gene product. TMHMM analysis results predicted that the N-terminal JHE-like region would reside outside of the cell, anchored on the cell membrane by the C-terminal transmembrane domain. From these results, it can be inferred that HzNV-2 captured the JHE gene from its lepidopteran host and evolved to alter its catalytic characteristics and specifically localize the Hz2V007 product at the surface of cells in infected tissue. When one considers the role of JH and JHE in controlling development and reproduction of insects, Hz2V007 might be a key gene responsible for the malformation of reproductive tissue functioning to regulate JH titers and gene expression in these tissues of the infected hosts. 

Hz2V012 and Hz2V015 encoded inhibitor of apoptosis (IAP) homologues. The IAP gene was first found in baculoviruses suppressing apoptosis of virus infected host cells [61]. The baculovirus IAPs have 1~3 baculovirus IAP repeat (BIR) conserved domains, and inhibit apoptosis by acting as inhibitors of the caspase family of protease enzymes. Baculovirus IAPs also contain a RING-finger motif and a zinc-binding site in their C-terminal region. The RING-finger motif is not required for anti-apoptotic activity [62,63]; however, it is important for regulation of IAPs by ubiquitin ligase [64,65]. Hz2V012 and Hz2V015 (termed IAP 1 and IAP 2) were 187aa and 182aa long peptides, respectively. These were both much shorter than other cellular or baculovirus IAPs. The best match of IAP 1 was the *Trichplusia ni* (cabbage looper) NPV IAP gene (E = 7 × 10^−25^), and the best match of IAP 2 was *Adoxophyes orana* GV IAP 3 (E = 1 × 10^−26^). IAP-1 had two BIR domains without a RING‑finger motif, while IAP 2 had one BIR domain with a C-terminal RING-finger motif. These are interesting results because most of the baculovirus IAPs have two BIR domains with one RING-finger motif at the C-terminal region. The exact role of the IAP genes of HzNV-2 still needs to be confirmed experimentally.

Hz2V023 had total of 12 characteristic transmembrane domains and showed homology to the major facilitator superfamily (MFS) conserved domain (E = 5 × 10^−10^). MFS is a family of transporter genes found ubiquitously in living organisms, which code for single-polypeptide carrier proteins involved in the uptake or efflux of small molecules. The primary functions of the MFS is uptake of sugar, and drug efflux [66]. Hz2V023 showed the best match to *A. aegypti* adenylate cyclase (E = 1 × 10^−135^).

Hz2V034 had homology to the guanosine monophosphate kinase (GMPK) motif at position 57–96 aa. The similarity was relatively low (E = 1 × 10^−3^), but the conserved catalytic motif of GMPK, G‑X2-G-X-G-K was found at position 61–67 aa as G-M-S-C-S-G-K with one aa mismatch at position 64 aa. GMPK catalyzes the reversible transfer of the terminal phosphoryl group of ATP and GMP to yield ADP and GDP [67]. This is an important step in the biosynthesis of GTP. GMPK also plays an important role in the recycling of the secondary messenger cGMP [68]. Hz2V034 showed best similarity to the hypothetical protein of MBV (E = 4 × 10^−5^).

Hz2V039 had homology to the baculovirus 19K protein gene (AcMNPV ORF96) which codes for a protein of unknown function. The 19K gene homologues are commonly found in baculoviruses, and Hz2V039 showed best similarity to GbNV ORF87 (E = 9 × 10^−9^). Hz2V059 and Hz2V073 encoded HzNV-1 *p34* and *p51* late gene homologues, respectively. Originally, *p34* and *p51* genes, which were detected from 8 to 24 hr post infection, were found in a cDNA library of HzNV-1 infected Tn-368 culture cells [28,69].

Hz2V068 encoded a zinc-dependent matrix metalloprotease (ZnMc_MMP) conserved domain (E = 2 × 10^−38^) and hemopexin-like repeat (HX) domains (E = 6 × 10^−26^) at the C-terminal flanking region. MMPs are zinc and calcium dependent enzymes, which are synthesized as proenzymes in connective tissues [70]. MMPs play an important role in cellular differentiation and morphogenesis being responsible for pericellular proteolysis of the extracellular matrix and other cell surface molecules. The activities of MMPs are regulated by a tissue inhibitor of metalloprotease (TIMP). [71]. The HX domain is often found in MMPs and their TIMPs. The signature of the MMP active site, HEXXHXUGUXH (U = bulky hydrophobic aa), was found at position 412–122 aa of Hz2V068, and putative TIMP-binding sites were found at the position 346–434 aa region. A possible cleavable signal peptide for secretion was also found at position 1–18 aa. Hz2V068 showed best similarity to *Acyrthosiphon pisum* (aphid) MMP (E = 3 × 10^−69^). Baculoviruses also have a metalloprotease, known as enhancin. Enhancin is incorporated in the occlusion body derived virus (ODV) and is thought to aid in the infection pathway via the degradation of intestinal mucin, a major protein component of the insect peritrophic membrane [72]. Hz2V068, however, shares a greater homology with cellular MMPs than with the baculoviral enhancins, which lack the hemopexin domain and a signal peptide. 

Hz2V096 showed homology to AcMNPV ORF81 (*ac81*). The *ac81* homologue is found in all baculoviruses, and is hence considered a baculovirus core gene [73]. A recent study of BmNPV ORF67, another homologue of Ac81, revealed that although its exact function is not known, it is a non-structural, late protein, detected in the cytoplasm of infected cells interacting with the cellular protein actin A3 [74]. Hz2V096 showed the best match to GbNV ORF14 (E = 6 × 10^−14^) and relatively low similarity to other baculovirus *ac81* homologues; however, the C-terminal transmembrane domain and the positions of cysteine residues common to Ac81s were conserved. Hence Ac81 homologues and Hz2V096 probably share similar secondary structure and perform similar functions during virus replication.

Hz2V099 showed homology to a prokaryotic acetylesterase (Aes) homologue. Aes is a member of esterase/lipase superfamily, and the Aes conserved domain was detected in Hz2V099 with relatively low similarity (E = 9 × 10^−4^). The Aes gene was first found in *Escherichia coli* and showed homology to lipase and esterase. Functional studies of the Aes protein demonstrated it had acetylesterase activity [75] and functioned in the control of a transcriptional activator [76].

Hz2V110 showed homology to the serine/threonine protein kinase (S_TKc) conserved domain (E = 1 × 10^−28^). Serine/threonine protein kinase catalyzes phosphorylation of serine and threonine residues, a major process in regulating cellular functions, especially protein phosphorylation in signal transduction [77].

### 2.9. Comparison of HzNV-2 and Baculovirus Genome Contents

A total of 13 complete baculovirus genome sequences have been compared, and 30 conserved core genes were found to be present in all baculoviruses [73]. These genes were organized into six categories based on their functions; replication, transcription, virus entry, structural proteins, auxiliary, and unknown. A total of 16 HzNV-2 genes found to share homology with the some of these baculovirus conserved core genes (Table 3). A total of 20 HzNV-1 genes have been reported to share homologies with baculovirus core genes (2). The HzNV-2 was found to contain homologues of all of these HzNV-1 genes however, no ORFs having homology with the 4 baculovirus core genes p47, ac68, vp39 and p33 were found in the HzNV-2 genome.

Among the DNA replication related baculovirus core genes, the most essential ones, DNA polymerase and helicase, were found in HzNV-2. However, LEF 1, DNA primase [78], and LEF 2, the LEF 1 associated protein [79], were not found in HzNV-2. Also *hrs*, the characteristic palindromic structures in baculovirus genomic DNA that function as transcriptional enhancers [80] and/or as origins of replication, were not found in HzNV-2. The absence of *lef-1*, *lef-2*, and *hrs* demonstrate that HzNV-2 possibly has a different viral DNA replication initiation mechanism than that of baculoviruses.

Five of the six conserved baculovirus core genes involved in transcription were found in HzNV-2. These five genes were *lef-4*, *lef-8*, and *lef-9*, which are the subunits of the baculovirus RNA polymerase, and LEF-5 and VLF-1, which are the late gene transcriptional factors of the baculovirus. The only conserved baculovirus gene involved in transcription that was not found in HzNV-2 was *p47*, one of the RNA polymerase subunits with unknown function [30]. It is interesting that HzNV-2 has baculovirus RNA polymerase and transcription factor homologues because having an alpha-amanitin resistant RNA polymerase is the one of the most distinctive features of the baculovirus compared with other DNA viruses [81]. Therefore, the presence of conserved homologues in HzNV-2 related to the transcription mechanism of baculoviruses supports the hypothesis that HzNV-2 and baculoviruses possibly evolved from a common ancestor.

Another group of conserved baculovirus core genes are the genes related to virus entry. The *p74*, *pif-1*, *pif-2*, and *pif-3* genes which are the *per os* infectivity factors, belong to this group. In the HzNV‑2 genome, all four of the *per os* infectivity related genes were found, while the fusion protein gene *p47* was not. This is a very interesting result since the primary route of transmission of HzNV-2 is not *per os* but rather by mating or through transovarial transmission. The similarity of *p74* and the *pif* genes to the HzNV-2 homologues were very low, so it is possible that these genes evolved in HzNV-2 to target reproductive tissue cells instead of the midgut cells. 

The baculovirus fusion protein, P47 is thought to play a role in systemic viral infections [82], The hymenopteran baculoviruses, *Neodiprion sertifer* NPV [83], and *Neodiprion lecontei* NPV [84], which also lack a fusion protein, replicate only in the midgut cells and do not exhibit systemic infection. This localized pathology compares with that of HzNV-2 in terms of non-systematic and tissue-specific infection. Hence, it is possible that the progeny virus of HzNV-2 in the target tissue could spread by cell surface specific binding of virus utilizing P74 and PIFs and not systemically via the fusion protein as do baculoviruses. 

HzNV-2 shares three of the eight baculovirus conserved genes coding for structural proteins, *odv-e56*, *vp91*, and *38K*, a capsid protein gene. ODV-e56 and VP91 are the virus structural proteins, while 38K is related to nucleocapsid assembly. The other five baculovirus conserved genes, *odv-e27*, *gp41*, *p6.9*, *vp39*, and *vp1054*, were not found in HzNV-2. GP41, P6.9, and VP39 are structural proteins which compose the nucleocapsid. VP1054 is responsible for nucleocapsid assembly and directly interacts with VP39 and 38K [84]. Only the 38K homologue was found in HzNV-2. The ODV-E27 is a structural protein and is considered a viral cyclin. It arrests host cells at the G_2_/M phase of the cell cycle with continuing viral DNA replication [85]. The absence of an *odv-e27* homologue in HzNV-2 might be expected since the pathogenesis of this virus in infected reproductive tissue is thought to occur with the development of reproductive tissue during the late larval and early adult stages of host insects.

Baculovirus alkaline exonuclease (alk-exo) while categorized as an auxiliary gene, has both 5'-3' exonuclease and endonuclease activity, as well as the ability to form complexes with the single strand binding protein and LEF 3. Alk-exo enzyme participates in dsDNA repair and is possibly responsible for the maturation of virus DNA in addition to maintenance of virus genome integrity [86,87]. An alk‑exo homologue was not found in HzNV-2, perhaps due to HzNV-2’s use of different DNA replication and repair mechanisms. Of the six baculovirus conserved genes, *ac68*, *ac81*, *ac92*, *ac96*, *ac109* and *ac142*, with unknown function, only *ac81* and *ac96* were found in HzNV-2.

**Table 3:** Comparison of conserved baculovirus core genes and HzNV-2.

Gene Function	Genes present in HzNV-2	Genes absent in HzNV-2
Replication	*dnapol* (*ac65*), *helicase* (*ac95*)	*lef-1* (*ac14*), *lef-2* (*ac6*)
Transcription	*lef-4* (*ac90*), *lef-5* (*ac99*), * lef-8* (*ac50*), *lef-9* (*ac62*), * vlf-1* (*ac77*)	*p47* (*ac40*)
Virus entry	*p74* (*ac138*), *pif-1* (*ac119*), *pif-2* (*ac22*), *pif-3* (*ac115*)	*ld130* (*ac23*)
Structural	*p91* (*ac83*), *38K* (*ac98*), * odv-e56* (*ac148*)	*odv-e27* (*ac144*), *gp41* (*ac80*), * p6.9* (*ac100*), *vp39* (*ac89*), * vp1054* (*ac54*)
Auxiliary		*alk-exo* (*ac133*)
Unknown	*ac81*, *19K* (*ac96*)	*ac68*, *ac92*, *ac109*, *ac142*

**Figure 5:**
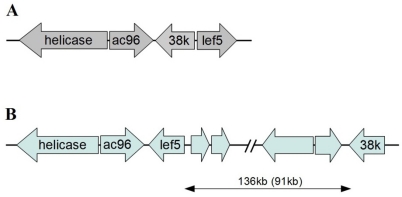
Comparison of the conserved core cluster of a baculovirus and corresponding region in HzNV-2. (**A**) Gene arrangement in the conserved core cluster of baculovirus; (**B**) Gene arrangement around HzNV-2 gene. The relative positions and orientations of *helicase* and *ac96* are the same in both baculovirus and HzNV-2, however, HzNV-2 has *lef-5* on the opposite DNA strand and *38 K* at 136 kb downstream (also 91 kb upstream) of the *helicase* gene.

Another characteristic of baculovirus conserved genes is a core gene cluster, which is a set of four genes (*helicase*, *ac96*, *38K*, and *lef-5*) found in the same relative position and direction in all known baculovirus genomes. This conserved gene cluster is very distinctive since extensive genome rearrangements are believed to have occurred in the evolution of baculoviruses [73]. HzNV-2 has a baculovirus core gene cluster-like region; however, it was observed to be different from that found in baculoviruses. The location and orientation of *helicase* and *ac96* were the same as in the baculovirus gene cluster, however *lef-5* was on the opposite strand, and orientation compare to its position in the baculovirus core cluster. The most distinctive difference was the location of *38k* gene. In baculoviruses, *38k* is located between the *ac96* and *lef-5* gene on the opposite DNA strand. The HzNV-2 *38k* homologue, however, is located 91 kb downstream of the *helicase* gene (Figure 5). This result may again reflect the divergence of HzNV-2 and baculoviruses during evolution from a common ancestor.

### 2.11. Phylogenetic Analysis

**Figure 6:**
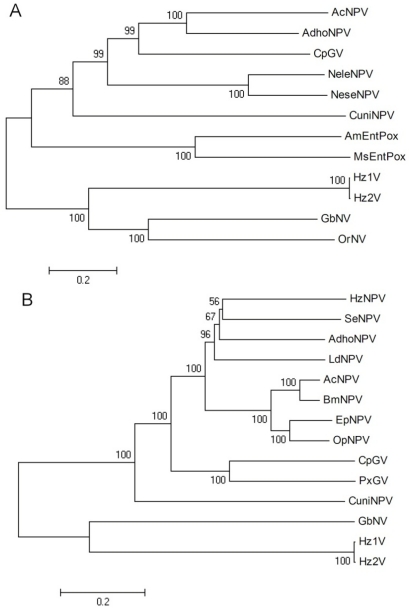
Phylogenetic analysis of selected insect DNA viruses and HzNV-2. (**A**) DNA polymerase; (**B**) combined sequence analysis *of p74*, *pif-1*, *pif-2* and *pif-3*; Multiple sequence alignments were performed by MUSCLE, and then the tree was inferred using neighbor-joining in MEGA4. The robustness of the tree was tested using bootstrap analysis (1000 replicates). The percent values are presented at the nodes. Organisms included in this analysis, with abbreviated names: *Autographa californica* NPV (AcNPV), *adoxophyes honmai* NPV (AdhoNPV), *Amsacta moorei* entomopoxvirus (AmEntPox), *Bombyx mori* NPV (BmNPV), *Culex nigripalpus* NPV (CuniNPV), *Cydia pomonella* granulovirus (CpGV), *Euproctis pseudoconspersa* NPV (EpNPV), *Gryllus bimaculatus* nudivirus (GbNV), *Lymantria dispar* MNPV (LdMNPV), *Melanoplus sanguinipes* entomopoxvirus (MsEPV), *Neodiprion lecontei* NPV (NeleNPV), *Neodiprion sertifer* NPV (NeseNPV), *Orgyia pseudotsugata* NPV (OpNPV), *Oryctes rhinoceros nudivirus* (OrNV), *Plutella xylostella* GV (PxGV).

DNA polymerase is one of key phylogenetic characteristics of virus taxonomy, therefore, a phylogenetic analysis was performed using this gene to assess the relationship of HzNV-2 to other insect DNA viruses. For this analysis, eleven DNA polymerase sequences were chosen from NPVs, GVs, entomopoxviruses, and NVs. A multiple sequence alignment was performed by MUSCLE, and a phylogenetic tree was built by neighbor-joining using MEGA4. The result demonstrated that Hz2V018 (the HzNV-2 DNA polymerase) grouped with GbNV, OrNV, and HzNV-1 DNA polymerase, while the baculovirus and entomopoxvirus DNA polymerases have monophyletic lineages (Figure 6A). This result showed that as expected, HzNV-2 DNA polymerase is more closely related to other NV DNA polymerases than to those of other insect viruses.

A similar approach was applied to the virus entry related protein genes. Although the similarities between these viruses are relatively low, *p74* and the *pif* genes are well-conserved genes in baculoviruses and NV. A possible explanation might be a diverging evolution of the *p74* and *pif* genes from an ancestral virus during host range expansion. The attachment and entry of viruses into cells is a key process in replication and is obligatory for horizontal transmission between susceptible hosts. The conservation of genes involved in this process across these different dsDNA viruses of insects (2), suggests that they share features of this process which have been maintained during their evolution. A total of 14 sets of *p74*, *pif-1*, *pif-2*, and *pif-3* genes chosen from baculoviruses and NV were combined for phylogenetic analysis. As with the DNA polymerase tree result, HzNV-2 grouped with other NV while the baculoviruses had a monophyletic lineage. This result demonstrated that the *p74* and *pif* genes of HzNV-2 are more closely related to those of NV and more distantly related to those of baculoviruses (Figure 6B). This result suggest that while there are some common elements in the process used by these viruses to enter host cells, and that NVs may share an aspect of this pathway which is different from that used by baculoviruses. Similar phylogenic analysis was performed in the genome analysis of the MdSGHV with results similar to those presented here (Figures 6A,B) [46].

## 3. Experimental Section

### 3.1. Source of Viruses

HzNV-2 viral particles were injected into newly emerged female moths from a laboratory colony of healthy *H. zea*, as outlined by Rallis and Burand [6]. After mating with healthy male moths, eggs laid by the injected female moths were collected from the mating chambers, then transferred into hatching chambers. Newly hatched larvae were reared on an artificial diet in environmental chambers under a light:dark cycle of 16:8 hours at 26 °C and upon emergence, progeny individuals were examined for signs of HzNV-2 infection, as described by Raina and Adams [88] and Hamm *et al*. [5]. Infected female moths with a visible, viral plug were collected as the source of viruses. The virus in the plugs were suspended in phosphate buffered saline (PBS) (136 mM NaCl, 1.4 mM KH2PO4, 8 mM Na2HPO4, 2.6 mM KCl, pH7.3), and used for virus particle preparation.

### 3.2. Virus Purification and DNA Extraction

The crude virus preparation in PBS, collected from the plugs, was layered on a sucrose gradient (25–55%), and centrifuged at 22,000 rpm at 4 °C for 1 h using a Beckman SW28 rotor. The purified virus band in the sucrose gradient was collected, diluted in TE buffer (10 mM Tris-HCl, 1mM EDTA, pH 8.0) or PBS, and centrifuged to pellet the purified virus particles. The purified virus particles were then suspended in TE or PBS and stored at −20 °C for DNA extraction.

The purified virus particles were incubated at 37 °C for 1 h with protease K at 1 mg/mL and sodium dodecyl sulfate (SDS) at 1% to solublize the virus envelope and structural proteins. The viral DNA was extracted twice with phenol, chloroform, and isoamylalcohol (25:24:1), followed by DNA precipitation with 0.3 M sodium acetate and 100% ethanol at −80°C for 1 hour. The extracted viral DNA was centrifuged at 12,000 rpm for 15 min at 4 °C, washed three times with 70% ethanol, air‑dried, resuspended in TE and stored at −20°C.

### 3.3. DNA Sequencing

Random DNA fragments of 1.5 to 6.5 kbp were obtained by partial enzymatic digestion with *Tsp*509I (New England Biolabs). The DNA fragments were cloned into the *Eco*RI site of pUC19 plasmid, and grown in *Escherichia coli* DH10B cells. Plasmids containing virus DNA fragments were purified by an alkaline lysis method. The fragments were sequenced from both ends of the cloning site with M13 forward and reverse primers using the dideoxy chain termination method [89] on a PRISM 3700 automated DNA sequencer (PE Biosystems). Chromatogram traces were base-called with PHRED and assembled with PHRAP [90,91]. Confirmatory assemblies were performed with the TIGR [92] and CAP3 [93] sequence assembly computer programs. The HzNV-2 virus DNA was sequenced to an average of 13-fold coverage at each base position. 

### 3.4. Sequence Analysis

Open reading frames (ORFs) longer than 60 amino acids (aa) with a methionine start codon were evaluated for coding potential using the Hexamer [94] and Glimmer [95,96] computer programs. Protein homology searches were performed using the BLAST, PSI-BLAST [97], FASTA [98], and HMMER [99] computer programs with the databases: PROSITE, Pfam, Prodom, and GenBank [100]. Global sequence alignment was performed using rVISTA [101]. Conserved domain searches were performed using the SMART [102] and CD-Search [103] computer programs with Swiss-Prot and CDD [104] databases. CGC [105], EMBOSS [106], Psort [107], TMHMM [108,109], SignalP 3.0 [110], and ANTHEPROT [111] computer programs were used for general analysis, signal peptide prediction, transmembrane domain prediction, and physical characterization of proteins. Phylogenetic analysis was performed by using ClustalX [112], MUSCLE [113], and MEGA4 [114].

## 4. Summary and Conclusions

We report here the complete nucleotide sequence and the annotation of the genome of the NV HzNV-2. This analysis showed HzNV-2 and HzNV-1 to have 93% sequence identity. Based on the high degree of sequence identity between these two NVs, it now appears that the IMC-Hz-1 ovarian cell line, from which HzNV-1 was originally isolated [115,116], was established from the ovaries of *H. zea* moths persistently infected with a NV which resembled HzNV-2. If this were the case, it is highly unlikely that females with grossly malformed reproductive tissues, characteristic of HzNV-2 infected insects, would have been selected for use in establishing an ovarian cell line. This possibility along with the absence of any observed pathology in HzNV-1 infected insects, suggest that genes responsible for the pathology caused by HzNV-2 may be missing in the HzNV-1 genome [11]. In this study, a total of 16 ORFs were identified in the HzNV-2 genome, that were not previously found in HzNV-1. Unfortunately, none of these 16 ORFs were determined to share sequence homology with any genes of know function, and therefore could not be directly associated with the pathology observed in HzNV-2 infected insects. Nonetheless, the genetic similarity between HzNV-1 and HzNV‑2 indicates that these two viruses are very closely related, having a very recent, common ancestor.

In a recent study, 20 HzNV-1 genes were found to share homologies with baculovirus core genes (2). Homologues of all of these HzNV-1 genes were found in the HzNV-2 genome however, there were no ORFs in HzNV-2 found to have homology with the 4 baculovirus core genes *p47*, *ac68*, *vp39* and *p33*. Interestingly, Hz1V075, which was found to be homologues to the OrNV *p47* was also reported to be homologous to OrNV *lef-9* (2). In our analysis, Hz2V063 was found to be homologous to Hz1V075 and MBV *lef-9,* but not to any *p47* ORFs. If Hz2V063 and Hz1V075 both have activity found associated with the two separate ORFs encoded by baculoviruses and other NVs, then this would suggest an important point of divergence of HzNVs from other N*Vs.*

An interesting feature of the HzNV-2 genome is the presence of a large number of cellular gene homologues. The HzNV-2 genome contains 12 genes which have homology to cellular genes. The majority of the cellular gene homologues in HzNV-2 are related to nucleic acid metabolism and DNA replication; 9 of the 12 cellular genes found in HzNV-2 are TS, dUTPase, DHFR, RR1, RR2, dNK, SHMT and ligase. The three other HzNV-2 genes found to more closely resemble host genes than viral genes (Hz2V023, Hz2068 and Hz2V007) are thought to code for proteins which could be involved in the regulation of molecular processes in the infected host leading to cell proliferation and tissue malformation observed in the reproductive structures of infected insects. 

It was interesting to find that HzNV-2 coded for a major facilitator superfamily (MFS) protein gene (Hz2V023). MFS genes are found ubiquitously in living cells [66] and have never before been identified in a viral genome. These transmembrane proteins are involved in the movement of small molecules, in and out of cells and could act to facilitate the enhanced metabolism required for the cell proliferation observed in HzNV-2 infected reproductive tissues. 

Hz2V068 which encodes both an MMP domain and an HX domain could also play an important role in altering the pattern of cell differentiation and morphogenesis. It is possible that this viral gene product could function in the proteolysis of the extracellular matrix and cell surface molecules, altering cell differentiation during HzNV-2 replication, resulting in the malformation of reproductive tissues during their development in infected insects. 

It was very surprising to find that HzNV-2 contains, Carboxylesterase (Hz2V007) which shares significant homology with *A. gambiae* JHE, suggesting that Hz2V007 codes for a protein that may have JHE activity. In holometabolous insects such as *H. zea* and *Drosophila*, the developmental process of molting is controlled by two hormones, the steroid, 20-hydroxyecdysone (ecdysone) and the sesquiterpenoid, juvenile hormone (JH). These two hormones work in concert to regulate gene expression during molting, directing insect growth and tissue development [117]. The predicted JHE activity of Hz2V007, coupled with increased cellular metabolic functions resulting from Hz2V023, MFS activity and tissue remodeling by Hz2V068, coded MMP could all contribute to malformation of virus infected tissues and the unique pathology observed in HzNV-2 infected moths. It has not yet been determined how each of these novel virus genes function, but we believe that it is the expression of these genes, along with several of the other cellular gene homologues identified in the HzNV-2 genome, which are responsible for the pathology and unique biology of this insect pathogenic virus.
